# Levonorgestrel-Releasing Intrauterine System vs. Usual Medical Treatment for Menorrhagia: An Economic Evaluation Alongside a Randomised Controlled Trial

**DOI:** 10.1371/journal.pone.0091891

**Published:** 2014-03-17

**Authors:** Sabina Sanghera, Tracy Elizabeth Roberts, Pelham Barton, Emma Frew, Jane Daniels, Lee Middleton, Laura Gennard, Joe Kai, Janesh Kumar Gupta

**Affiliations:** 1 Health Economics Unit, University of Birmingham, Birmingham, United Kingdom; 2 School of Clinical and Experimental Medicine, University of Birmingham, Birmingham, United Kingdom; 3 Birmingham Clinical Trials Unit, University of Birmingham, Birmingham, United Kingdom; 4 Division of Primary Care & National Institute for Health Research, University of Nottingham, Nottingham, United Kingdom; Groningen Research Institute of Pharmacy, Netherlands

## Abstract

**Objective:**

To undertake an economic evaluation alongside the largest randomised controlled trial comparing Levonorgestrel-releasing intrauterine device (‘LNG-IUS’) and usual medical treatment for women with menorrhagia in primary care; and compare the cost-effectiveness findings using two alternative measures of quality of life.

**Methods:**

571 women with menorrhagia from 63 UK centres were randomised between February 2005 and July 2009. Women were randomised to having a LNG-IUS fitted, or usual medical treatment, after discussing with their general practitioner their contraceptive needs or desire to avoid hormonal treatment. The treatment was specified prior to randomisation. For the economic evaluation we developed a state transition (Markov) model with a 24 month follow-up. The model structure was informed by the trial women's pathway and clinical experts. The economic evaluation adopted a UK National Health Service perspective and was based on an outcome of incremental cost per Quality Adjusted Life Year (QALY) estimated using both EQ-5D and SF-6D.

**Results:**

Using EQ-5D, LNG-IUS was the most cost-effective treatment for menorrhagia. LNG-IUS costs £100 more than usual medical treatment but generated 0.07 more QALYs. The incremental cost-effectiveness ratio for LNG-IUS compared to usual medical treatment was £1600 per additional QALY. Using SF-6D, usual medical treatment was the most cost-effective treatment. Usual medical treatment was both less costly (£100) and generated 0.002 more QALYs.

**Conclusion:**

Impact on quality of life is the primary indicator of treatment success in menorrhagia. However, the most cost-effective treatment differs depending on the quality of life measure used to estimate the QALY. Under UK guidelines LNG-IUS would be the recommended treatment for menorrhagia. This study demonstrates that the appropriate valuation of outcomes in menorrhagia is crucial.

## Introduction

Menorrhagia, or heavy menstrual bleeding, places a considerable burden on healthcare resources, with around 6% of women per year consulting their general practitioners [1]. The condition can be defined as “Excessive menstrual blood loss which interferes with a woman's social, emotional, physical and material quality of life” [2]. Treatment is prompted predominantly by a woman's subjective assessment of interference in her quality of life, rather than solely by clinical assessment of volume of blood loss [3]. Women may change, or cease treatment, according to their perception of effectiveness, and relative to their contraceptive needs.

Historically, women often progressed quickly to a surgical solution; either hysterectomy, resulting in the permanent cessation of bleeding and sterility, or since the 1990s, endometrial ablation, which uses electrical or thermal energy to destroy the endometrium, causing amenorrhea in 34% of women [4]. Non-hormonal and hormonal medical treatments are now available as first line therapy for women presenting with menorrhagia in primary care.

In 2007, the National Institute of Health and Care Excellence (NICE) introduced guidelines for the Levonorgestrel-releasing intrauterine system (LNG-IUS) to be used for treatment of menorrhagia based on limited evidence on cost-effectiveness [2]; [5]. Nine other small trials have compared LNG-IUS to non-hormonal and hormonal treatments, showing reduction in menstrual blood loss but these did not consider cost-effectiveness [6]; [3].

To our knowledge, no direct cost-effectiveness comparison of LNG-IUS against usual medical treatment has been reported. We undertook an economic evaluation as part of the ECLIPSE trial (**E**ffectiveness and **C**ost-effectiveness of **L**evonorgestrel-containing **I**ntrauterine system in **P**rimary care against **S**tandard tr**E**atment for menorrhagia), a pragmatic, multicentre, randomised trial, comparing the clinical and cost-effectiveness of LNG-IUS against usual medical treatment in the primary care setting. Further, as impact on quality of life is the primary outcome measure in menorrhagia, quality of life was measured using two different instruments including EQ-5D and SF-6D. Both instruments are accepted measures for valuing health related quality of life, although NICE's preferred measure is EQ-5D [7]. Since treatment is prompted by women's own assessment of interference on quality of life it was considered appropriate to explore the influence on the cost-effectiveness results of using both instruments.

## Methods

We conducted a model-based economic evaluation in the form of a cost-utility analysis, based on an outcome of cost per quality adjusted life year (QALY) alongside the ECLIPSE trial [8]. The QALY outcome encapsulates quality and quantity of life into a single metric. The analysis was carried out from a UK National Health Service (NHS) perspective in a primary care setting and provides an assessment of the difference in costs and QALYs between interventions over a 24-month time horizon. A second analysis will be carried out at the 5-year time point. A societal perspective to include private costs to women was considered but deemed not to be feasible given the resource constraints for data collection.

### Participants and trial design

The ECLIPSE trial, which found LNG-IUS to be more effective than usual treatment, is reported in detail elsewhere [8]. Briefly, 571 women with menorrhagia from 63 UK centres were randomised between February 2005 and July 2009. Women between 25 and 50 years of age presenting to their general practitioner (GP) with menorrhagia, occurring over at least three consecutive cycles, provided written informed consent to participate. The definition of menorrhagia used is consistent with that used in the RCOG guidelines and was the basis of the clinical paper published in the New England Journal of Medicine [8]; [9]. Women were excluded if they intended to become pregnant over the next 5 years, were taking hormone replacement therapy or tamoxifen, had intermenstrual or post-coital bleeding or examination suggestive of fibroids (abdominally palpable uterus equivalent in size to 10–12 weeks' gestation) or other pathologies, or had contraindications to, or a preference for, LNG-IUS or usual medical treatments. Women were allocated to a treatment group by telephone or web-based central randomisation service. Women were randomised to having a LNG-IUS fitted, or usual medical treatment, chosen by the GP and the woman based on contraceptive needs or desire to avoid hormonal treatment.

Usual medical treatment options included mefenamic acid, tranexamic acid, norethisterone, a combined estrogen/progestogen or progestogen only oral contraceptive pill (any formulation), or methoxyprogesterone acetate injection [2]; [9] The particular medical treatment was specified prior to randomisation. Treatment review by the GP at 6 weeks and 3 months was recommended. Subsequently, treatments could be changed or discontinued as per usual practice (e.g. due to perceived lack of benefit, side effects, change in contraception need, referral for endometrial ablation or hysterectomy) [2]; [9]. Treatment changes reported by women were confirmed with the GP.

Ethical approval was obtained from the National Research Ethics Service Committee South West - Exeter and clinical trial authorization from the Medicines and Healthcare Regulatory Authority. Written consent was obtained from the participants. The name of the trial registry is ISRCTN and the number is ISRCTN86566246.

### Model

We developed a state transition (Markov) model in place of the typical trial-based economic analysis to comprehensively account for the changes in quality of life that occurred whilst the women were taking these treatments.

As outlined later, data on utilities (or quality of life) were collected at baseline, 6 months, 1 year and 2 years. The trial data showed that, due to its non-curative nature, women were changing their treatment, more frequently within this time, to identify the best method for managing menorrhagia, and this process had an influence on their quality of life. The analysis does not lend itself to a regression framework because patients change between different health states on a monthly basis and quality of life was not measured at that frequency. It would therefore be inappropriate to infer QALYs in a typical trial based analysis from the quality of life scores at the time point they happen to be taken in the trial. The most suitable method to capture changes in quality of life, occurring throughout the trial, and provide a robust cost-utility analysis, was to represent these experiences as health states in a decision model, which follows the process of management of menorrhagia used in the ECLIPSE trial. Therefore, quality of life values were attached to the health states. A measure of change in utility from baseline and the endpoint of the trial, as in a typical trial-based analysis which does not use a decision model, would not accurately capture the health states that women had experienced throughout the time span of the trial. Furthermore, a typical trial-based analysis, without a decision model, would not comprehensively capture the time spent in health states or the associated repetitive costs and resource use. The decision model, based on trial data, provides a more realistic explanation of the utility pathway, providing information that can be synthesised with other data and projected forward. Specifically, a Markov model appropriately combines data for each of the pathways and takes account of the cyclical and repetitive nature of events and facilitates a simulation approach. All parameters used in the model were based on the trial data.

**Figure 1:**
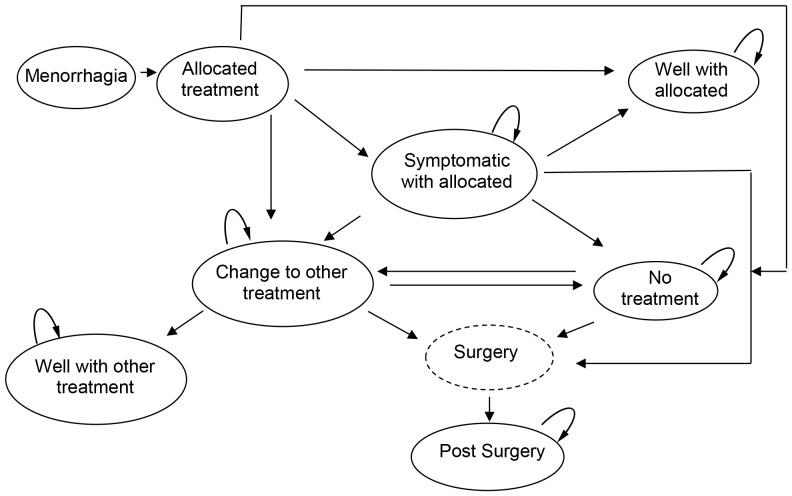
Clinical pathway for LNG-IUS and usual medical treatment.

The model structure was informed by clinical input and the pathways followed by the women in the ECLIPSE trial. *Figure 1* presents the clinical pathways and the progress of the two cohorts of women in the ECLIPSE trial who were randomised to LNG-IUS or usual medical treatment. A monthly time cycle is used as this represents the clinically meaningful changes observed in treatment and resource use. The following assumptions were made and developed based on clinical expertise from practising primary care and gynaecology clinicians, in addition to standard treatment protocol. The assumptions were agreed prior to conducting the analysis.

*Model assumptions*

- A woman is ‘well’ with the allocated treatment if she does not change or stop treatment. Some of these women may not be ‘well’ but are coping with treatment, and the utility values for the ‘well’ with allocated treatment state reflect this.
- A woman who is ‘well with LNG-IUS’ or ‘well with usual medical treatment’ cannot spontaneously become ‘symptomatic’.
- Based on the data, if in the first cycle, women move from the allocated treatment to an alternative state other than ‘well’, it is assumed they either move to the ‘change to alternative treatment’ or the ‘no treatment’ state. We assume that they do not move to the ‘symptomatic’ state in the first cycle because insufficient time has elapsed to establish this and so it is assumed they changed for other reasons.
- From the second cycle onwards, if women change from their allocated treatment they do not go to ‘well’ but to the ‘symptomatic’ state and move on from there.
- For the transition to the ‘surgery’ state, data were collected on whether a woman had ablation or hysterectomy, but not the precise technique e.g. thermal balloon endometrial ablation or microwave endometrial ablation. Data on the weighted likelihood of surgery undertaken were taken from a previous study [10]. In the model we assumed that if a woman in the trial has endometrial ablation, it will be her first ablation and we apply the cost for first line endometrial ablation techniques.
- Once a woman in the model has changed from the allocated treatment, it is not possible for her to move back to the allocated treatment.
- We assumed that if a woman ‘changes to the other treatment’, she must spend at least one cycle in ‘change to other treatment’ before she can move to ‘well with other treatment’. This is required because it will take at least one menstrual cycle for any effect to become apparent.

### Outcome measures

Outcome measures were collected using both EQ-5D and SF-36 at baseline prior to randomisation, then by post at 6 months, 1 year and 2 years post-randomisation. The booklet questionnaire, given to women in the trial, contained the generic EQ-5D-3L questionnaire, which measures the impact of treatment on broader aspects of health related quality of life [11]. SF-36 was converted into SF-6D using the algorithm [12]. Utility values for each state in the model were calculated by averaging the EQ-5D or SF-6D values for each woman in the given state at any given time.

Utility values for the individual states were calculated by averaging the EQ-5D (SF-6D) values obtained by each woman in the given state at any given time. For example if a woman is randomised to LNG-IUS and then does not change treatment she is considered to be in the ‘well’ state for the remainder of the analysis, as outlined in the model assumptions previously. Therefore all of the woman's utility values collected at 6 months, 1 year and 2 years will be assigned to the ‘well’ health state in the model. Similarly if a woman is initially ‘symptomatic with LNG-IUS’ and then moves to ‘no treatment’ at 2 years, the utility values for 6 months and 1 year will be assigned to ‘symptomatic’ and the utilities for 2 years assigned to the ‘no treatment’ health state. This method was used to derive the utility values because the utility for the state is important, not the values associated with the individual woman's journey, as decision models are a reflection of the typical population.

### Costs and Resource use

Costs were collected from a UK NHS perspective. Data on healthcare resource use, including GP or gynaecologist consultations, were collected from women alongside other outcome measures. The general healthcare costs for both groups included healthcare staff costs and the cost of the interventions. An LNG-IUS fitting was estimated to take 20 minutes (informed by clinical experts within trial team), require both a GP and nurse to be present and also require disposable consumables. Treatment review by the GP was assumed to last 10 minutes (informed by clinical experts within trial team). Staff costs were calculated using nationally recognised reference costs [13]. The costs of standard medical treatment and LNG-IUS were estimated from the British National Formulary [14]. Cost data on surgical interventions were taken from a previously published study and inflated to 2011 [10]. As recommended by NICE a discount rate of 3.5% was applied to both costs and utilities as the model time horizon is beyond 1 year [7]. All costs are reported in 2011 prices in UK (£) sterling using the UK hospital and community health services index [13]. Tables 1–3 present the data used in the analysis.

**Table 1:** Cost data used in the analysis.

	Unit cost	Source
**LNG-IUS**		
Consultation (GP 10 mins)	£26.67	Curtis 2011/expert opinion
***Insertion***		
GP (20 mins)	£53.33	Curtis 2011/expert opinion
Practice nurse (20 mins)	£17.00	Curtis 2011/expert opinion
Device cost	£88.00	BNF 62
Sterile pack (insertion)	£21.63	NICE (inflated to 2011)
***Discontinuation***		
GP (10 mins)	£26.67	Curtis 2011/expert opinion
Practice nurse (10 mins)	£8.50	Curtis 2011/expert opinion
Sterile pack (removal)	£3.77	NICE (inflated to 2011)
***Follow-up***		
6 week review: (GP 10 mins)	£26.67	Curtis 2011/expert opinion
3 month: (GP 10 mins)	£26.67	Curtis 2011/expert opinion
**Usual medical treatment**		
***Progestogen (Cerazette)***	£8.68	BNF 62
***Tranexamic acid (cyclokapron)***	£14.30	BNF 62
***Mefenamic acid (Ponstan)***	£15.72	BNF 62
***Norethisterone***	£2.18	BNF 62
***Combined oral contraceptive (microgynon)***	£2.82	BNF 62
***Methoxyprogesterone acetate injections (Depo-provera)***	£6.01	BNF 62
Consultation: (GP 10 mins)	£26.67	Curtis 2011/expert opinion
3 or 6 month review (GP 10 mins)	£26.67	Curtis 2011/expert opinion
Discontinuation (GP 10 mins)	£26.67	Curtis 2011/expert opinion
**Surgery**	£1720.18	Weighted cost from trial

All costs are presented in 2011 (£) sterling.

**Table 2:** Health state utility data used in the model.

Health State	EQ-5D value	PSA Distribution (EQ-5D)	SF-6D Value#	PSA Distribution (SF-6D)#	Source
***LNG-IUS***					
LNG-IUS	0.756	Beta (653, 211)	0.597	Beta (10204, 6883)	ECLIPSE trial
Well with LNG-IUS	0.98	Beta (1169, 297)	0.598	Beta (17912, 12061)	ECLIPSE trial
Symptomatic with LNG-IUS	0.744	Beta (130, 45)	0.589	Beta (3464, 2418)	ECLIPSE trial
Change to usual medical treatment	0.817	Beta (20, 5)	0.596	Beta (1066, 723)	ECLIPSE trial
Well with usual medical treatment	0.714	Beta (66, 26)	0.594	Beta (2032, 1390)	ECLIPSE trial
No treatment	0.785	Beta (70, 19)	0.604	Beta (2108, 1380)	ECLIPSE trial
Surgery	0.620	Linked to post surgery	0.430	Linked to post surgery	ECLIPSE trial
Post-surgery	0.827	Beta (59, 12)	0.574	Beta (330, 245)	ECLIPSE trial
***Usual medical treatment***					
Usual medical treatment	0.714	Beta (514, 206)	0.603	Beta (9892, 6519)	ECLIPSE trial
Well with usual medical treatment	0.728	Beta (528, 197)	0.592	Beta (9664, 6647)	ECLIPSE trial
Symptomatic with usual medical treatment	0.756	Beta (311, 100)	0.606	Beta (5168, 3359)	ECLIPSE trial
Change to LNG-IUS	0.694	Beta (49, 21)	0.627	Beta (2494, 1484)	ECLIPSE trial
Well with LNG-IUS	0.801	Beta (282, 70)	0.595	Beta (4069, 2766)	ECLIPSE trial
No treatment	0.766	Beta (223, 68)	0.586	Beta (3548, 2509)	ECLIPSE trial
Surgery	0.619	Linked to post-surgery	0.454	Linked to post surgery	ECLIPSE trial
Post-surgery	0.825	Beta (64, 14)	0.606	Beta (2136, 1391)	ECLIPSE trial

Utility values are rounded to 3 decimal places. α and β values for the PSA distribution are rounded to the nearest whole number. LNG-IUS; levonorgestrel-releasing intrauterine system, PSA; probabilistic sensitivity analysis.

# Values used in sensitivity analysis 4.

**Table 3:** Probability parameters used in the analysis.

Probability Parameters	Probability	PSA distribution
***LNG-IUS***		
LNG-IUS to well with LNG-IUS	0.639	(182, 103) Dirichlet
LNG-IUS to symptomatic with LNG-IUS	0.253	(72, 213)
LNG-IUS to change to usual medical treatment	0.067	(19, 266)
LNG-IUS to no treatment	0.042	(12, 73)
Remain Well with LNG-IUS	1	Fixed
Symptomatic with LNG-IUS to well with LNG-IUS	0	Fixed
Remain symptomatic with LNG-IUS	0.907	(700, 72) Dirichlet
Symptomatic with LNG-IUS to change to usual medical treatment	0.035	(27, 745)
Symptomatic with LNG-IUS to no treatment	0.041	(32, 740)
Symptomatic with LNG-IUS to surgery	0.017	(13, 759)
Remain change to usual medical treatment	0.708	(109, 45) Dirichlet
Change to usual medical treatment to well with usual medical treatment	0.208	(32, 122)
Change to usual medical treatment to no treatment	0.045	(7, 147)
Change to usual medical treatment to surgery	0.039	(6, 148)
Remain well with usual medical treatment	1	Fixed
No treatment to change to usual medical treatment	0	(1, 547) Dirichlet
Remain no treatment	0.984	(540, 8)
No treatment to surgery	0.016	(10, 538)
Surgery to post surgery	1	Fixed
Remain post surgery	1	Fixed
***Usual medical treatment***		
Usual medical treatment to well with usual medical treatment	0.402	(115, 171) Dirichlet
Usual medical treatment to symptomatic with usual medical treatment	0.566	(162, 124)
Usual medical treatment to change to LNG-IUS	0.007	(2, 284)
Usual medical treatment to no treatment	0.024	(7, 279)
Remain Well with usual medical treatment	1	Fixed
Symptomatic with usual medical treatment to well with usual medical treatment	0	Fixed
Remain symptomatic with usual medical treatment	0.901	(1474, 162) Dirichlet
Symptomatic with usual medical treatment to change to LNG-IUS	0.049	(80, 1556)
Symptomatic to no treatment	0.040	(65, 1571)
Symptomatic to surgery	0.010	(17, 1619)
Remain change to LNG-IUS	0.603	(120, 79) Dirichlet
Change to LNG-IUS to well with LNG-IUS	0.312	(62, 137)
Change to LNG-IUS to no treatment	0.045	(9, 190)
Change to LNG-IUS to surgery	0.040	(8, 191)
Remain well with LNG-IUS	1	Fixed
No treatment to change to LNG-IUS	0.001	(1, 852) Dirichlet
Remain no treatment	0.992	(846, 7)
No treatment to surgery	0.007	(6, 847)
Surgery to post surgery	1	Fixed
Post surgery to post surgery	1	Fixed

α and β values for the PSA distribution are rounded to the nearest whole number. LNG-IUS; levonorgestrel-releasing intrauterine system, PSA; probabilistic sensitivity analysis.

In cases where women were prescribed a combination of the usual medical treatments, a weighted average of the cost is taken. Similarly, repeat prescription costs were calculated based on the average weighted cost of repeat prescriptions in the ‘change to usual medical treatment’ state of the LNG-IUS arm. As the most commonly prescribed usual medical treatments involved GP review for effectiveness at 3 months, it was assumed that this occurs at 3 months.

## Analysis

An incremental cost-utility analysis provides information on the difference in costs and QALYs between LNG-IUS and usual medical treatment and is reported in terms of incremental cost-effectiveness ratios (ICERs), as cost per QALY gained. If a treatment is less costly and generates a greater number of QALYs, dominance is said to occur. Analysis was by intention-to-treat to provide a pragmatic estimate of ICERs. The base case analysis and three deterministic sensitivity analyses were carried out using EQ-5D. An additional sensitivity analysis, repeating the base case and its three deterministic sensitivity analyses was carried out using SF-6D.

Uncertainty in the model was explored by conducting both deterministic and probabilistic sensitivity analysis. Population heterogeneity was not considered by assessing the cost-effectiveness according to population subgroups because the randomised nature of the trial should mean that there are no systematic differences between women in each treatment arm.

*Deterministic Sensitivity Analysis:*

1. We replaced the mean utility values for each state used in the base case by the median utility value. Previously some published studies used the median and not the mean value, which greatly impacts the cost-effectiveness results and is argued to be inappropriate [10]. The current analysis assesses the impact of using such values when primary data are collected.
2. We incorporated the assumptions used in the UK national guidelines costing template to replace the expert opinion and trial data that was used in the base case. This change applied to the clinical staff member present for the initial consultation, which used a practice nurse and was assumed to be 10 minutes for the initial consultation and insertion (GP in the base case for the initial consultation, and 20 minutes for the insertion with both GP and practice nurse), treatment review by a nurse at 6 weeks (GP was used in the base case) only for those with a LNG-IUS fitted and annual follow-up for both treatment groups thereafter (no annual follow-up in the base case) [2].
3. In the base case, we assumed that when an EQ-5D completion date and notification of change of treatment coincide, the EQ-5D value will belong to the subsequent state. In sensitivity analysis 3, we assigned the EQ-5D value to the state prior to the change.
4. We repeated the base case analyses and the three deterministic analyses (described in 1 to 3 above) but used SF-6D, instead of EQ-5D to generate QALYs.

The probabilistic sensitivity analysis simultaneously changes all relevant parameters in the model. For each parameter, a distribution is assigned and a value for each parameter is randomly drawn from the assigned distribution. This is repeated 1000 times and the range of incremental cost and QALY results for LNG-IUS and usual medical treatment are presented on the cost-effectiveness plane. We used these 1000 values to construct a cost-effectiveness acceptability curve (CEAC) to illustrate the probability of LNG-IUS being more cost-effective than usual medical treatment, across a range of monetary values that decision-makers may be willing to pay for an additional QALY. This was carried out using both EQ-5D and SF-6D.

## Results

Table 4 presents the base case and deterministic sensitivity analysis results using EQ-5D. The base case results show that LNG-IUS costs £100 more than usual medical treatment, as it costs £430 whilst usual medical treatment costs £330. However, LNG-IUS also generated 0.067 more QALYs than usual medical treatment as LNG-IUS generated 1.580 QALYs and usual medical treatment 1.513 QALYs. The base case results show that LNG-IUS generates £1600 per additional QALY when compared to usual medical treatment.

**Table 4:** Base case and deterministic sensitivity analysis results using EQ-5D.

	Total costs per intervention (£)	Total QALYs per intervention	Incremental cost effectiveness ratio (ICER) (v usual medical treatment)
**Summary of base case deterministic results**			
Usual medical treatment	330	1.513	1600
LNG-IUS	430	1.580	
*Mean Difference*	100	0.067	
**Deterministic sensitivity analysis 1** *			
Usual medical treatment	330	1.590	2030
LNG-IUS	430	1.643	
*Mean Difference*	100	0.053	
**Deterministic sensitivity analysis 2** #			
Usual medical treatment	340	1.513	1640
LNG-IUS	450	1.580	
*Mean Difference*	110	0.067	
**Deterministic sensitivity analysis 3** °			
Usual medical treatment	330	1.514	1560
LNG-IUS	430	1.582	
*Mean Difference*	100	0.068	

Cost are rounded to nearest 10. QALYs are rounded to 3 decimal places QALYS; quality adjusted life year, LNG-IUS; levonorgestrel-releasing intrauterine system, ICER; incremental cost-effectiveness ratio.

^*^Deterministic sensitivity analysis 1 =  Use median utility values.

# Deterministic sensitivity analysis 2 =  Use NICE assumptions.

**°**Deterministic sensitivity analysis 3 =  Assigning EQ-5D completion date utility for change treatment, if change treatment date is the same as EQ-5D completion date.

In deterministic sensitivity analyses 1, 2 and 3, which all used EQ-5D as per base case, the findings supported the base case results. However, the ICER in each analysis did differ (see Table 4). Sensitivity analysis 1 and 2 had a slightly less favourable effect, increasing the ICER to £2030 and £1640 per QALY gained respectively, whilst sensitivity analysis 3 resulted in a more favourable effect on the ICER with a reduction to £1510 per additional QALY.

**Figure 2:**
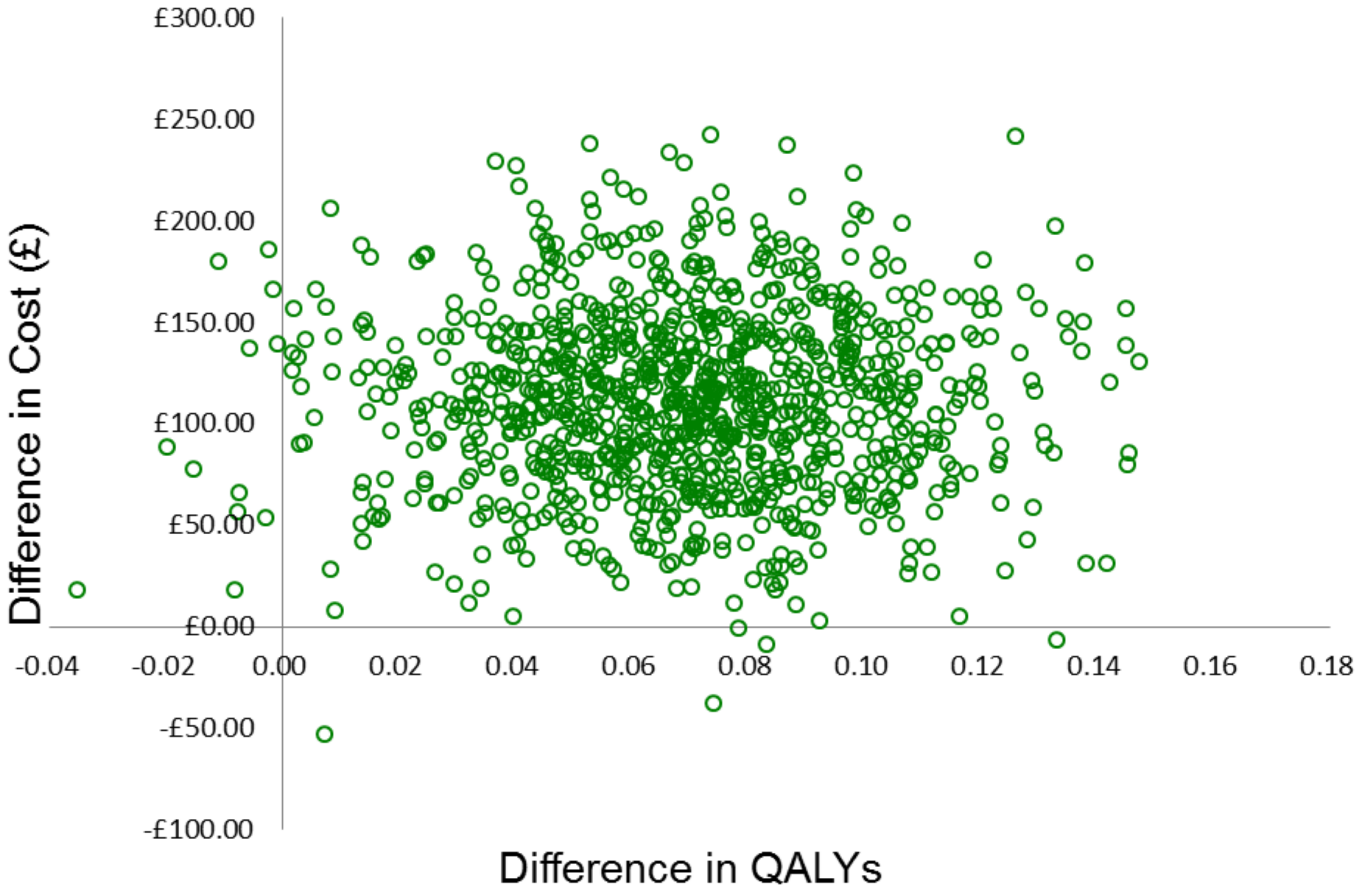
Results of the probabilistic sensitivity analysis (EQ-5D).

**Figure 3:**
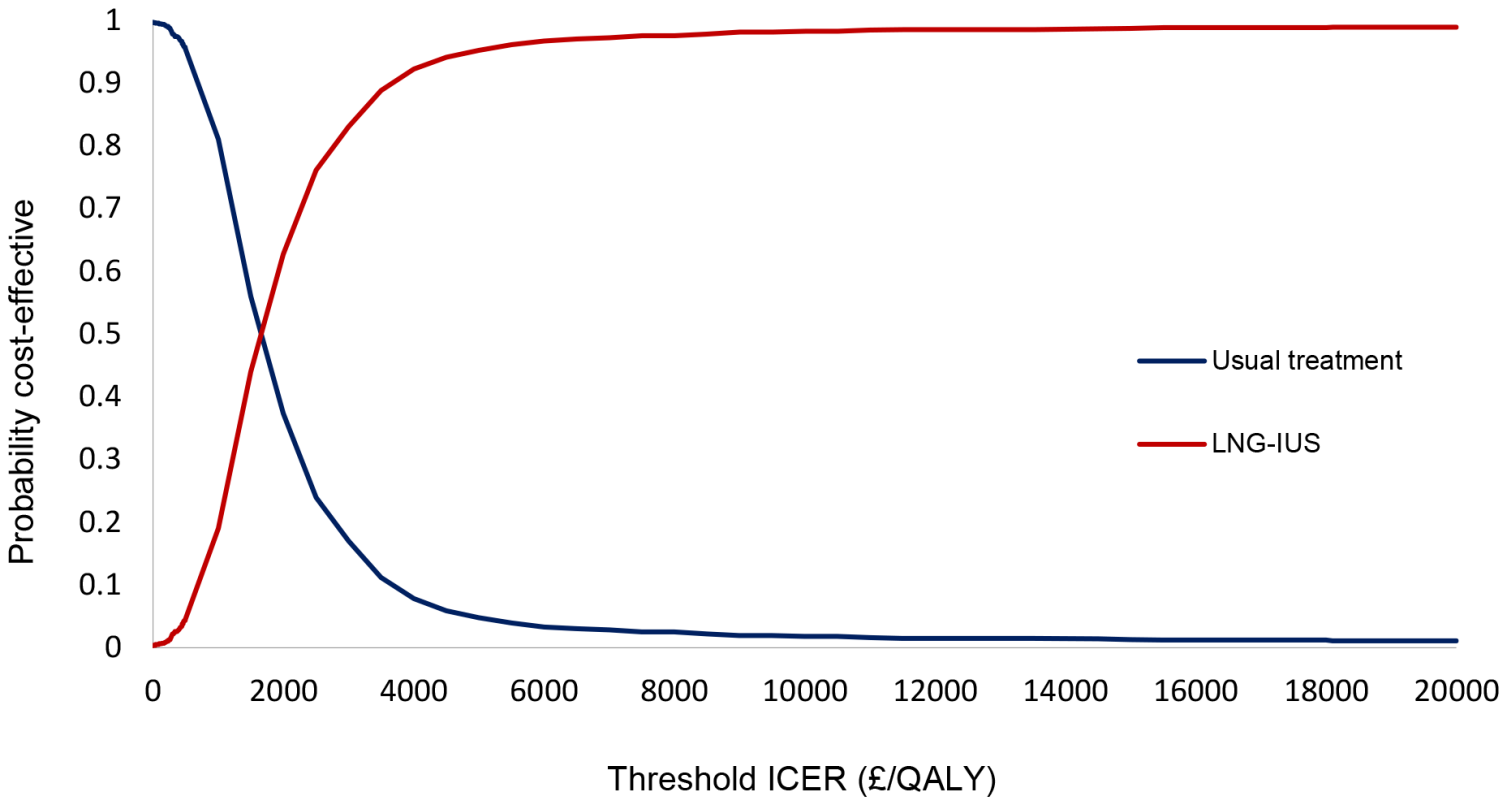
Cost-effectiveness acceptability curves for usual medical treatment and LNG-IUS using EQ-5D.

The results of the probabilistic sensitivity analysis (Figure 2) illustrate the distribution of the incremental costs and effects (EQ-5D) from 1000 Monte Carlo simulations. The uncertainty is then summarised in relation to the changes in the decision-makers threshold for considering an intervention cost-effective in Figure 3. It depicts the CEAC, which shows that from £2000 per QALY, LNG-IUS has a greater probability of being the more cost-effective intervention. This probability increases to over 90% at approximately £4000 per QALY.

In sensitivity analysis 4, where the analysis was repeated using SF-6D instead of EQ-5D, the results are presented in Table 5. Similar to the base case results LNG-IUS costs £100 more than usual medical treatment as it costs £430 whilst usual medical treatment costs £330. However, when SF-6D is used, usual medical treatment is shown to generate 0.002 more QALYs than LNG-IUS, as usual medical treatment generated 1.200 QALYs and LNG-IUS 1.198 QALYs. Therefore, it is shown that usual medical treatment dominates LNG-IUS. Usual medical treatment is shown to dominate in all but one of the analyses using SF-6D. The exception was where EQ-5D values were replaced by SF-6D for sensitivity analysis 3, in which case LNG-IUS was shown to be more effective than usual medical treatment and more expensive, generating an ICER of around £110,000.

**Table 5:** Sensitivity analysis 4 results using SF-6D.

	Total costs per intervention (£)	Total QALYs per intervention	Incremental cost effectiveness ratio (ICER) (v usual medical treatment)
**Summary of base case deterministic results**			
Usual medical treatment	330	1.200	Dominates
LNG-IUS	430	1.198	
*Mean Difference*	100	−0.002	
**Deterministic sensitivity analysis 4.1** *			
Usual medical treatment	330	1.215	Dominates
LNG-IUS	430	1.215	
*Mean Difference*	100	0	
**Deterministic sensitivity analysis 4.2** #			
Usual medical treatment	340	1.200	Dominates
LNG-IUS	450	1.198	
*Mean Difference*	110	−0.002	
**Deterministic sensitivity analysis 4.3** °			
Usual medical treatment	330	1.198	112,340
LNG-IUS	430	1.199	
*Mean Difference*	100	0.001	

Cost are rounded to nearest 10. QALYs are rounded to 3 decimal places. QALYS; quality adjusted life year, LNG-IUS; levonorgestrel-releasing intrauterine system, ICER; incremental cost-effectiveness ratio.

^*^Deterministic sensitivity analysis 1 =  Use median utility values.

# Deterministic sensitivity analysis 2 =  Use NICE assumptions.

**°**Deterministic sensitivity analysis 3 =  Assigning SF-6D completion date utility for change treatment if change treatment date is the same as SF-6D completion date.

**Figure 4:**
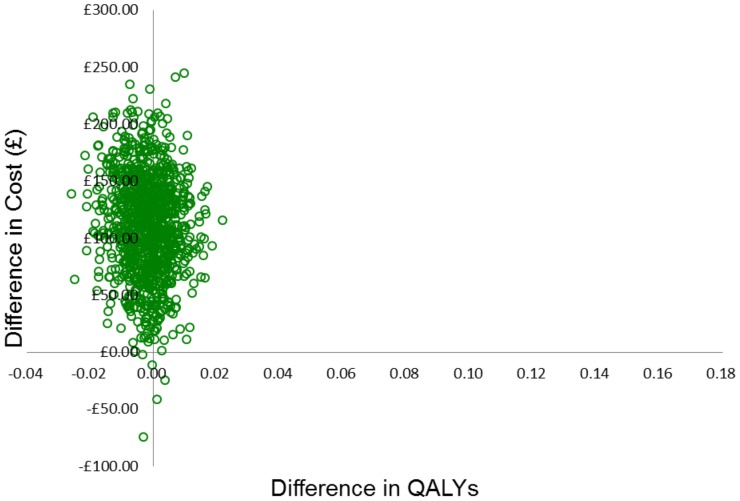
Results of the probabilistic sensitivity analysis (SF-6D).

**Figure 5:**
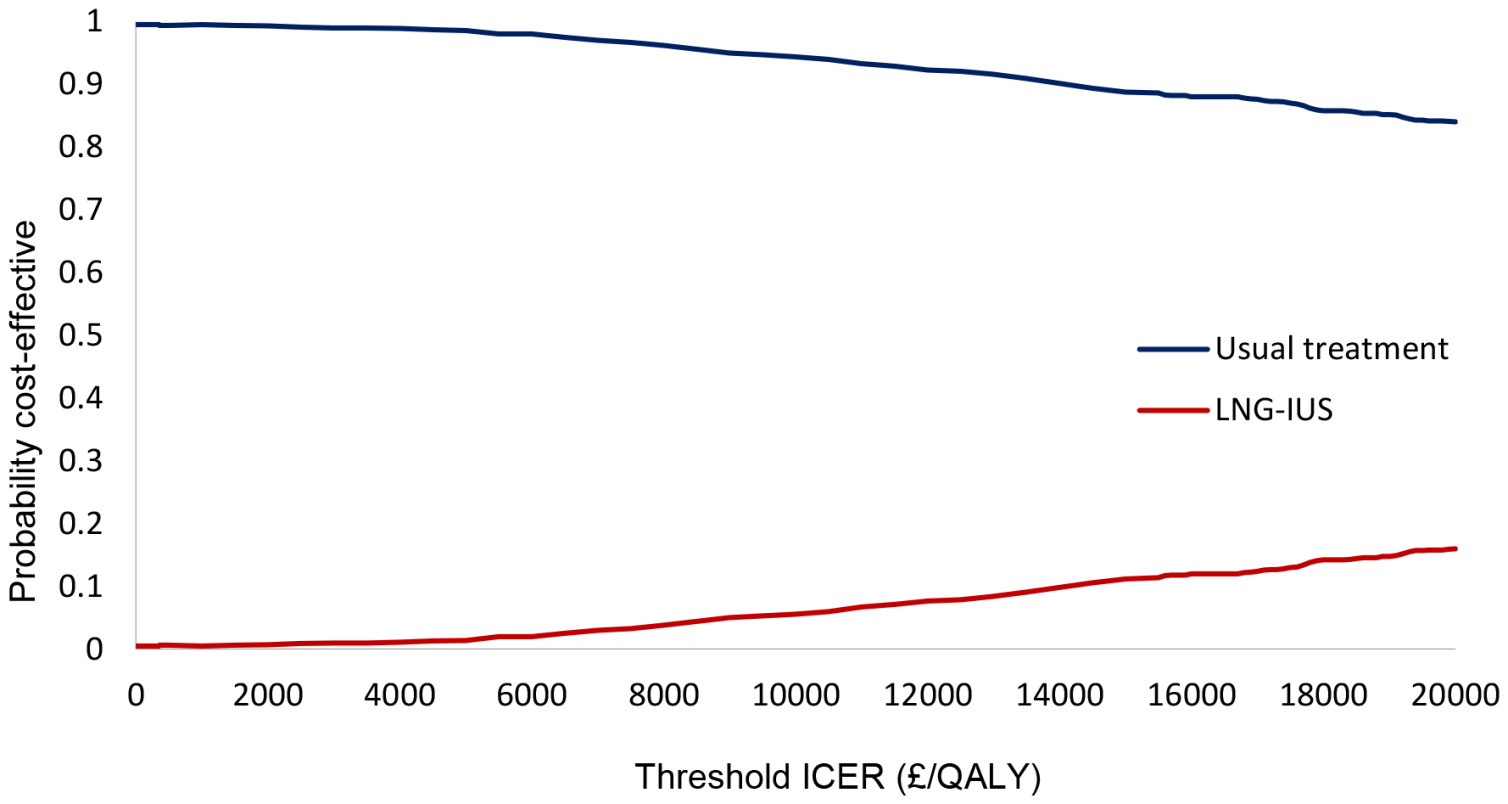
Cost-effectiveness acceptability curves for usual medical treatment and LNG-IUS using SF-6D.

The results of the probabilistic sensitivity analysis and the CEAC using SF-6D are presented in Figures 4 and 5. The CEAC shows that for any threshold willingness-to-pay per QALY, usual medical treatment has the greater probability of being the more cost-effective intervention. This probability is 100% at £0 per QALY and decreases to 90% at approximately £20,000 per QALY.

## Discussion

### Main findings

In the primary care setting, treating menorrhagia using LNG-IUS, costs more but is also more effective than usual medical treatment. The relative cost-effectiveness of LNG-IUS compared to usual medical treatment is £1600 per QALY. This means every additional QALY costs an extra £1600. The deterministic sensitivity analyses showed the uncertainty in this ICER to be in the range of £1,560–£2,030 for additional QALY gained. As the NICE guidelines recommend new interventions into practice if the ICER is below £20,000 per QALY, LNG-IUS would be considered cost-effective and recommended as the primary choice for women who require treatment, have no preference against contraception or intrauterine insertion, and no contraindication to LNG-IUS insertion.

However, the importance of selecting the most appropriate quality of life instrument is highlighted when the measure used to assess quality of life is changed in sensitivity analysis 4 from EQ-5D to SF-6D. In sensitivity analysis 4, where utility values from SF-6D are used rather than EQ-5D, the cost-effectiveness results differ. In contrast to the findings using EQ-5D, usual medical treatment is the more cost-effective intervention. Usual medical treatment was found to dominate LNG-IUS in the base case and the two deterministic sensitivity analyses. In the third deterministic analysis, the ICER for LNG-IUS was over £100,000 per QALY which is much higher than the £20,000/QALY threshold currently set by NICE, therefore usual medical treatment would be recommended for implementation in clinical practice.

The difference in the cost-effectiveness results derived by using the alternative measures to value quality of life has a considerable impact on the cost-effectiveness decision. The different measures did not just change the strength of cost-effectiveness of the same treatment, but the most cost-effective treatment itself changed. Therefore the recommendation to decision-makers would differ depending on the quality of life instrument used.

### Strengths and limitations

The strength of this cost-utility analysis is that it is based on data from the largest multi-centre randomised trial undertaken for menorrhagia. Since the treatment is aimed at managing the condition, the changes in both quality of life and costs throughout the woman's treatment journey are critical to the analysis and these are most appropriately captured by using the trial data to populate a model. The model structure was developed based on the women's pathway data from the trial and supported by the advice of expert clinicians. All assumptions were agreed by the team in the model development stage prior to analysis. A further strength is that all data on resource use and outcomes were collected prospectively alongside the trial and the economic evaluation was an integral component of the trial design from the outset.

Some limitations exist as a result of some of the assumptions required for the model. For instance it was assumed that women are ‘well’ if they do not change treatment. Nonetheless this assumption is mitigated by the fact that if women enter the ‘well’ state, but are not well, it will be reflected in the overall utility value for ‘well’.

A further potential limitation is that baseline differences in quality of life data at the outset of the trial were not adjusted to be the same. The initial EQ-5D score in the LNG-IUS group was 0.042 higher at the outset of the trial than in usual medical treatment and this difference is significant (p<0.05). These data are based on individuals who have been randomised, so it is assumed that this difference occurred by chance and it does not follow that this initial difference between groups would be sustained over the 2 year time horizon in the absence of treatment. Adjustment for baseline therefore risks imposing a difference at every point in time over the time period of analysis.

If we did adjust fully for the difference in baseline we would show LNG-IUS to be less effective than usual medical treatment, but in so doing, the assumption would be imposed that the difference would be maintained over the time horizon. This would not be an appropriate assumption since the probability of regression to the mean over time would be ignored. It is therefore acknowledged that the base case results might be slightly over optimistic but it is not clear how much of an adjustment, if any, would be appropriate.

### Comparison with other studies

To our knowledge, this is the first study to conduct a cost-utility analysis using prospectively collected primary data from a trial to directly compare LNG-IUS and usual medical treatment for women with menorrhagia. Other economic evaluations of LNG-IUS have been carried out, however none have drawn a comparison of LNG-IUS directly against usual treatment using primary data. Another primary study has shown LNG-IUS to be cost-effective, but the comparator was hysterectomy and the study is considered to have methodological flaws [15]; [10]. Other studies have compared alternative treatments using model based analyses and secondary data from reviews [2]; [16]; [17] although, the studies typically compared LNG-IUS, various oral treatments and various *surgical* techniques against one another. Two of these showed LNG-IUS to be the most cost-effective intervention [2]; [16]. The remaining study suggested that hysterectomy was the optimal intervention but the authors acknowledged that insufficient published data on the effectiveness of LNG-IUS were available at the time [17]. Although LNG-IUS is shown to be cost-effective against surgical interventions in these studies, it does not provide evidence for LNG-IUS being cost-effective against usual pharmaceutical treatment.

### Implications and Further Research

The results, based on the decision-maker recommended EQ-5D, provide clear evidence in support of the NICE guidelines that recommend LNG-IUS be considered the primary treatment for menorrhagia. The main objective of this study was to provide evidence to decision-makers on the cost-effectiveness of two treatments for menorrhagia, the primary results therefore are based on EQ-5D. However, as SF-6D data were also collected, the use of this measure was deemed worthy of exploration in the sensitivity analysis.

When SF-6D is used to generate QALYs, the main base case results are reversed and usual medical treatment is shown to be the more cost-effective intervention. Hence the recommendation to decision-makers differs depending on the outcome measure used. The conflicting findings suggest that these measures may be capturing different aspects of quality of life which clearly has an impact on the results. Reasons why these instruments produce different results have been further explored by other authors (Brazier et al [18] and Whitehurst and Bryan [19]) but this does not help to guide which instrument is more appropriate within the context of menorrhagia, particularly when the results are so sensitive to that choice. It may well be that neither instrument is appropriate, with their focus exclusively on health-related quality of life, and consideration needs to be given to alternative measures which is evidenced in a recent quality of life review [20]. Future research might appropriately explore alternative methods for measuring outcomes that are important to women and these might include outcomes which are not based solely on health related quality of life.

This manuscript reports the 2 year follow-up and was commissioned by the NIHR-HTA for an analysis at 2 years. As the condition is chronic and continues until menopause, further economic evaluations with longer term follow-up will be reported and published as and when they are available.
